# X-Ray Exposure Induces Structural Changes in Human Breast Proteins

**DOI:** 10.3390/ijms26125696

**Published:** 2025-06-13

**Authors:** Ren Jie Tuieng, Sarah H. Cartmell, Cliona C. Kirwan, Alexander Eckersley, Michael J. Sherratt

**Affiliations:** 1 Division of Cell Matrix Biology & Regenerative Medicine, School of Biological Sciences, Faculty of Biology, Medicine and Health, Manchester M13 9PT, UK; snrrjt@nus.edu.sg; 2 Singapore Nuclear Research and Safety Institute, National University of Singapore, Singapore 118415, Singapore; 3 Department of Materials, School of Natural Sciences, Faculty of Science and Engineering, The University of Manchester, Manchester M13 9PL, UK; sarah.cartmell@manchester.ac.uk; 4 The Henry Royce Institute, Royce Hub Building, The University of Manchester, Manchester M13 9PL, UK; 5 Division of Cancer Sciences, School of Medical Sciences, Faculty of Biology, Medicine and Health, University of Manchester, Oglesby Cancer Research Building, Manchester Cancer Research Centre, Wilmslow Road, Manchester M20 4BX, UK; cliona.kirwan@manchester.ac.uk; 6 The Nightingale Breast Cancer Unit, Wythenshawe Hospital, Manchester University NHS Foundation Trust, Manchester M23 9LT, UK; 7 Division of Musculoskeletal & Dermatological Sciences, School of Biological Sciences, Faculty of Biology, Medicine and Health, Manchester M13 9PT, UK; alexander.eckersley@manchester.ac.uk; 8 Manchester Cell-Matrix Centre, Faculty of Biology, Medicine and Health, Manchester M13 9PT, UK

**Keywords:** radiotherapy, X-rays, breast cancer, mass spectrometry, extracellular matrix, collagen

## Abstract

During radiotherapy, X-rays can deliver significant doses of ionising radiation to both cancerous and healthy tissue, often leading to undesirable side effects that compromise patient outcomes. While the cellular effects of such therapeutic X-ray exposures are well studied, the impact on extracellular matrix (ECM) proteins remains poorly understood. This study characterises the response of ECM proteins, including the major tissue components collagen I and fibronectin (FN), to X-ray doses similar to those used in clinical practice (50 Gy, as employed in breast radiotherapy, and 100 Gy), using a combination of gel electrophoresis, biochemical assays, and mass spectrometry-based peptide location fingerprinting (PLF) analysis. In purified protein solutions, 50 Gy X-ray exposure led to the fragmentation of constituent collagen I α chains. Irradiation of purified plasma FN (pFN) induced localised changes in peptide yields (detected by liquid chromatography and tandem mass spectrometry (LC-MS/MS) and PLF) and enhanced its binding to collagen I. In complex environments, such as newly synthesised fibroblast-derived ECM and mature ex vivo breast tissue, X-ray exposure induced peptide yield changes in not only collagen I and FN but also key basement membrane proteins, including collagen IV, laminin, and perlecan. Intracellular proteins associated with gene expression (RPS3, MeCP2), the cytoskeleton (moesin, plectin), and the endoplasmic reticulum (calnexin) were also found to be impacted. These X-ray-induced structural changes may impair the ECM integrity and alter cell–ECM interactions, with potential implications for tissue stiffening, fibrosis, and impaired wound healing in irradiated tissues.

## 1. Introduction

Human tissues are exposed to X-ray radiation during diagnostic imaging and radiotherapy [1]. Patients undergoing imaging or radiotherapeutic procedures are often exposed to X-rays with energies of kilo- to mega-electron volts in doses of ~0.4 mGy [2] (imaging) and 50 Gy (radiotherapy) [3]. Such radiotherapeutic exposures profoundly impact cell physiology, producing reactive oxygen species (ROS), mainly hydroxyl radicals [4,5], which oxidise proteins and produce double-stranded breaks in DNA, leading to cell death [6,7]. Whilst the aim of treatment is to target cancer cells, radiotherapy can also induce significant damage to surrounding healthy tissues, resulting in undesirable clinical side effects, including longer-term alterations in tissue extracellular matrices (ECM) [8,9]. In breast cancer radiotherapy, stromal tissues in the breast, skin, heart, and lungs receive substantial doses of ionising radiation [10,11]. Acute side effects include breast erythema [12] and moist desquamation [13], whilst chronic effects, such as skin thickening, muscle atrophy, pericarditis, coronary artery damage, and pulmonary and ECM fibrosis, may take months or years to manifest [8,9,14,15,16].

In tissues, ECM proteins provide biochemical and mechanical cues which influence cellular behaviour [17,18]. For example, the adhesive glycoprotein fibronectin (FN) mediates cell–ECM interactions, which, in turn, influence mechano-transduction pathways [19,20], whilst growth factors, including isoforms of transforming growth factor beta, bind to the small leucine-rich proteoglycans decorin and biglycan [21]. In pathological situations, the degradation and fragmentation of ECM proteins, such as collagen I, can liberate peptide matrikines, which modulate wound healing and inflammation processes [22,23]. Long-term studies on the effects of ionising radiation exposure have identified aberrant ECM remodelling [24,25], with therapeutic X-ray doses altering tissue biomechanical properties [24] and the biochemical environment [26]. These effects may be due either to direct X-ray exposure of ECM proteins or to indirect and chronic cell-mediated remodelling. Previous studies have established that exposure to high-dose ionising radiation (1000’s to 100,000’s of Gy) can directly cleave peptide bonds and impact collagen I structure [27,28], but these doses are not clinically relevant. We have recently shown that therapeutically relevant doses of X-ray radiation can directly affect the structure of purified collagen I monomers both in suspension and in fibril assemblies [29].

This study aimed to investigate the direct and immediate impact of X-ray exposure on ECM proteins in complex proteomes in the absence of subsequent cell-mediated matrix remodelling. We first characterised the effects of exposure to X-ray doses of 50 Gy (similar to some total therapeutic exposures) and 100 Gy in the following two simple model systems: purified collagen I and purified plasma FN (pFN). However, as the extracellular environment in tissues is complex and formed of multi-component assemblies, two further systems were also exposed to the same X-ray doses, as follows: (i) ECM-enriched, sample from cultured fibroblasts (fECM), which contained newly synthesised proteins, and (ii) human breast tissue, which contained a mature ECM. The breast tissue samples also facilitated studies of X-ray interactions with intracellular proteins. Conventional biochemical approaches (gel electrophoresis) were combined with mass spectrometry (LC-MS/MS)-based peptide location fingerprinting (PLF) analysis [30,31,32] to detect radiation-induced structural changes in proteins.

## 2. Results

### 2.1. 50 Gy and 100 Gy X-Ray Exposure Affects the Structure and Binding of Purified Collagen I and Fibronectin

**Figure 1:**
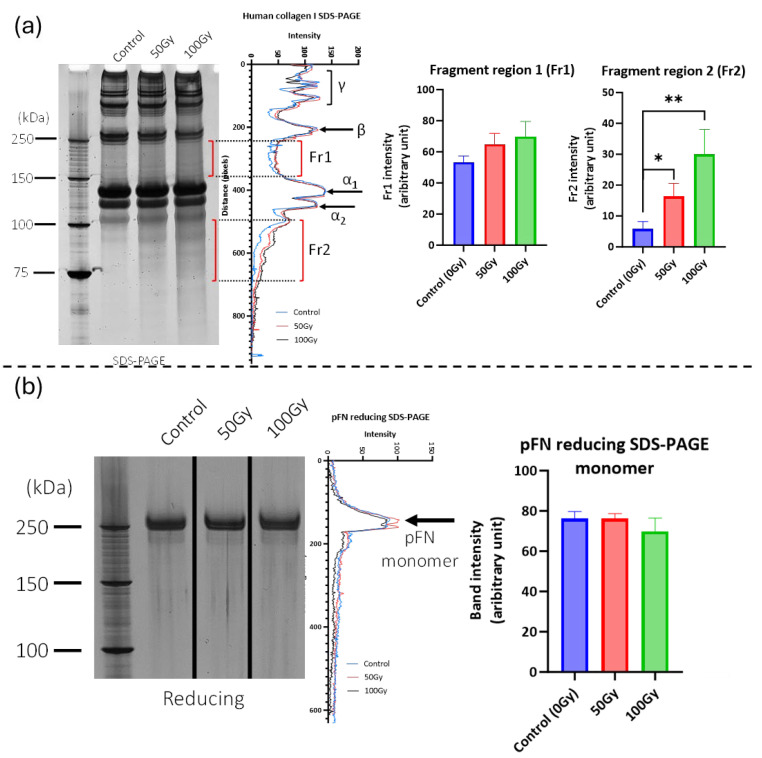
Effect of X-ray exposure on the electrophoretic profile of collagen I and pFN. (**a**) Exposure to both 50 Gy and 100 Gy X-ray doses induced significant dose-dependent fragmentation of the primary backbone of collagen I. In the Fr 1 region (between the β dimer and γ trimer bands), background-corrected staining intensity increased by 22% (50 Gy: *p* = 0.06) and 31% (100 Gy: *p* = 0.06). In the Fr 2 region (below the a2 band), staining intensity increased by 180% (50 Gy: *p* = 0.02) and 415% (100 Gy: *p* = 0.008). Statistical significance is represented by * (*p* < 0.05) and ** (*p* < 0.01) (**b**) No significant changes were detected in the electrophoretic mobility and staining intensity of the FN dimer following X-ray exposure (*n* = 3).

We have previously shown that X-ray exposure affects the structure of collagen I, fragmenting monomers in suspension and inducing changes in tryptic peptide profile (as assessed by PLF) in collagen I fibrils [29]. Here, using SDS-PAGE, we confirm that X-ray doses of 50 Gy and 100 Gy are able to fragment solubilised collagen I monomers (Figure 1a). In contrast, exposure to these same doses had no discernible effect on the SDS-PAGE profile of pFN (Figure 1b).

**Figure 2:**
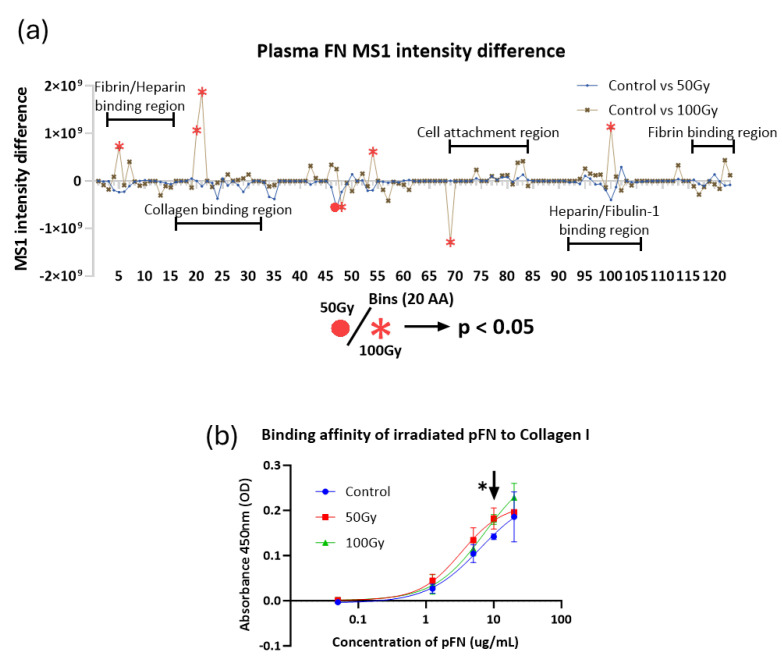
Impact of X-ray exposure on the structure and collagen I binding capability of pFN. (**a**) Using PLF, differences in peptide MS1 intensities along the pFN sequence were compared between control and treated groups (50 Gy and 100 Gy, *n* = 5). The pFN amino acid sequence was computationally divided into bins of 20 amino acids. Bins where MS1 intensities were significantly altered (*p* < 0.05) are marked in red (circle for 50 Gy, star for 100 Gy). Positive values indicate greater summed peptide intensities in control; negative values indicate greater intensities in treated samples; while zero values represent either no change in peptide yield or no peptides detected. More regions with significant differences were identified in 100 Gy-exposed pFN than 50 Gy-exposed, and these regions are associated with fibrin, heparin, and collagen-binding sites. (**b**) Solid-phase ELISA of pFN in suspension (non-irradiated and irradiated, *n* = 3) incubated with immobilised collagen I substrate. X-ray exposure increased pFN-binding affinity to collagen I at a concentration of 10 µg/mL, with 29% and 27% increases in absorbance for 50 Gy (*p* = 0.04) and 100 Gy (*p* = 0.006), respectively. A consistently higher binding affinity was observed for both 50 Gy- and 100 Gy-exposed pFN across 1–10 μg/mL pFN concentrations, although not statistically significant. Curve fitting was achieved with GraphPad Prism 9.0 software’s built-in sigmoidal 4-parameter logistic curve (4PL) with R2 values of 0.91 (Control), 0.96 (50 Gy), and 0.97 (100 Gy).

However, subsequent PLF analysis of X-ray-exposed pFN did detect regions with significant peptide yield changes (Figure 2a and Supplementary Figure S1). Exposure to a 50 Gy X-ray dose induced a significant change in peptide yield at a single location. However, exposure to 100 Gy induced changes in localised peptide yield at the same location and at a further five positions including the binding regions for cells, fibrin, heparin, fibulin-1, and collagen I. In order to test if the functionality of the X-ray-exposed collagen-binding site was affected, we conducted solid-phase ELISA experiments assessing the ability of irradiated pFN to bind to a surface-adsorbed collagen I substrate (Figure 2b). X-ray-exposed pFN was found to exhibit consistently higher binding affinity for collagen I than non-exposed pFN at concentrations of 1–10 ug/mL; although, this difference was only statistically significant (for both 50 Gy and 100 Gy treatments) at a concentration of 10 μg/mL.

### 2.2. X-Ray Exposure Alters Structure of Extracellular Proteins in Complex Matrices

Having shown that X-ray doses of 50 Gy and 100 Gy can affect the structure of purified proteins, we next investigated the impact of the same X-ray exposures on more complex systems, as follows: (i) a newly synthesised, cell culture-derived ECM (decellularised and hence ECM-enriched: fECM) and (ii) a mature tissue ECM (breast tissue).

#### 2.2.1. Fibroblast Cell Culture-Derived and Breast Tissue Samples Are Both Rich in ECM Proteins

**Figure 3:**
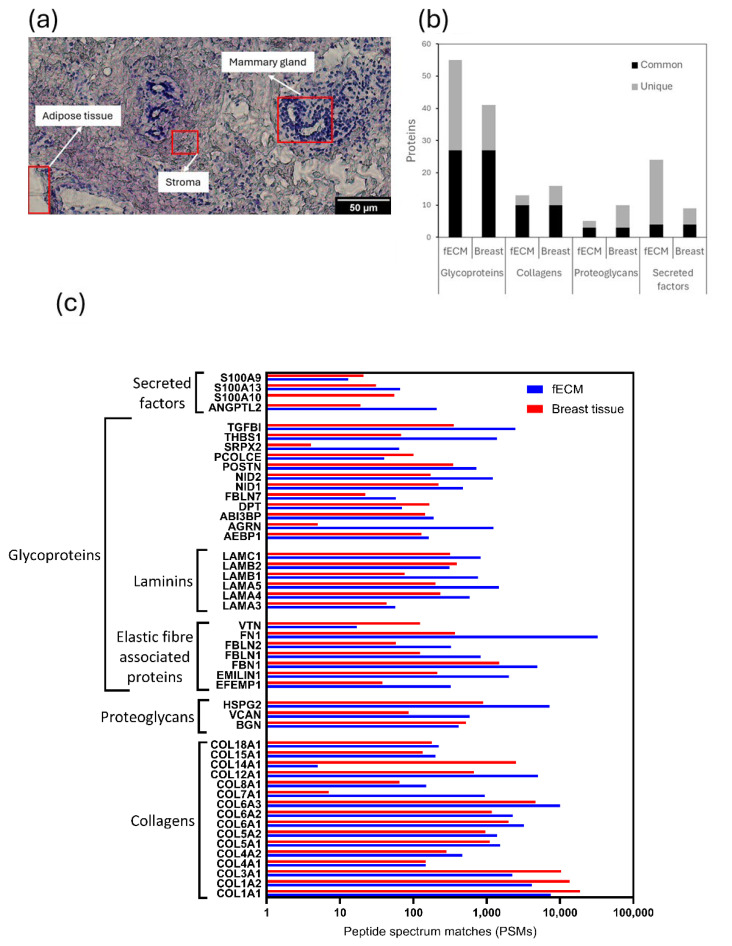
Comparison of cultured fibroblast (fCEM) and breast tissue extracellular matrix. (**a**) Representative breast tissue cryosection stained with Hematoxylin and Eosin Y (H&E). Regions corresponding to the three tissue types within breast are identifiable (annotated red boxes). (**b**) Abundance of unique and common ECM glycoproteins, collagens, proteoglycans, and secreted factors in fECM and breast tissue. (**c**) Respective abundances (approximated through counting peptide spectrum matches [PSMs]) of 48 individual ECM proteins common to both fECM (*n* = 5) and the breast tissue sample (*n* = 1).

The relative protein compositions of complex fECM and breast tissue (containing glandular, stroma, and adipose tissue areas: Figure 3a) samples were compared by LC-MS/MS. A total of 1097 and 738 proteins (both extracellular and intracellular) were detected in fECM and breast tissue respectively. With reference to MatrisomeDB, 85 proteins in fECM and 67 in breast tissue were classified as core matrisome ECM proteins (collagens, ECM glycoproteins, and proteoglycans) or secreted factors. These are hereinafter referred to as ‘ECM proteins’. Glycoproteins were the most commonly identified ECM protein category with 27 found in both fECM and breast tissue, compared with 10 collagen types (Figure 3b). While there were substantial numbers of unique glycoproteins in fECM compared with breast tissue samples, the identities of the collagens were largely conserved. Within the cohort of 48 proteins common to both matrices were representative proteins from major ECM assemblies including fibrillar and non-fibrillar collagens, elastic fibre associated proteins, and basement membrane components (Figure 3c).

#### 2.2.2. X-Ray Exposure Affects the Structure of Multiple ECM Proteins in Both Cell- and Tissue-Derived Proteomes

**Figure 4:**
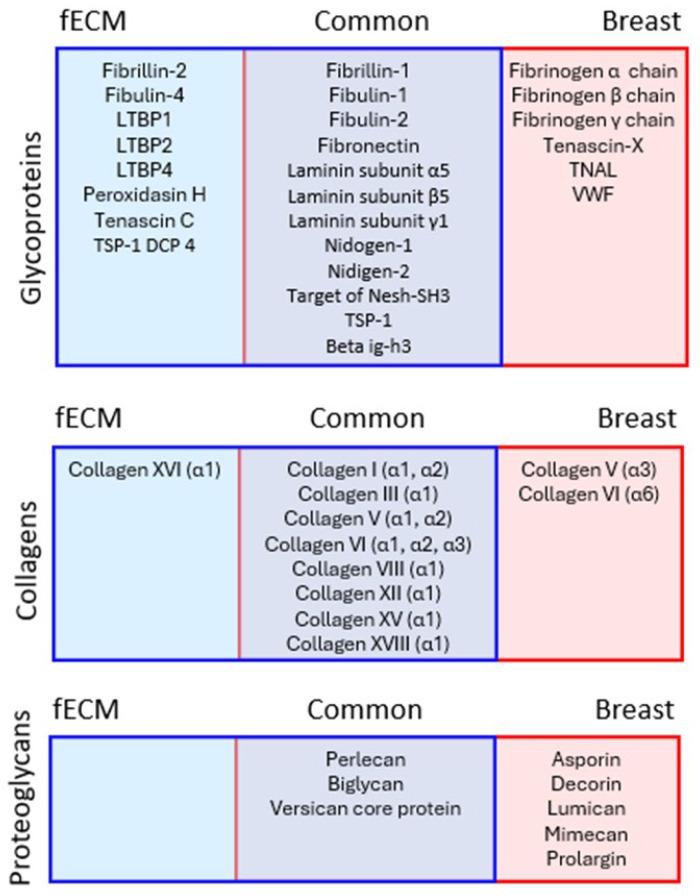
X-ray labile proteins in cultured fibroblast and breast tissue matrices. Proteins found only in fECM, only in breast tissue, or in both, that were identified by PLF analysis as structurally compromised by X-ray exposure. Each of these proteins contained at least one bin within their protein sequence with a statistically significant change in peptide MS1 intensity after exposure to 50 Gy or 100 Gy of X-ray exposure.

A PLF analysis of all ECM proteins identified 28 glycoproteins, nine collagens (most represented by multiple alpha chains), and eight proteoglycans, which were X-ray labile in either fECM, breast tissue, or both (Figure 4). Each X-ray labile protein contained at least one bin within its amino acid sequence with a statistically significant (*p* < 0.05) change in peptide yield after exposure to either 50 Gy or 100 Gy X-ray doses. These proteins included fibrillar (I, III, and V), fibril-associated (XII), and network forming (VI and VIII) collagens [33] and small, leucine-rich proteoglycans, such as decorin and biglycan, which play an important role in collagen fibril assembly [34,35]. Elastic fibre-associated proteins (fibrillins, fibulins, and LTBPs), basement membrane components (laminins and niodgens), and adhesive glycoproteins (FN) were also identified as X-ray labile, suggesting that X-ray exposure is likely to affect many different ECM functions.

#### 2.2.3. Impact of X-ray on Collagen I and Fibronectin in Complex Matrices

**Figure 5:**
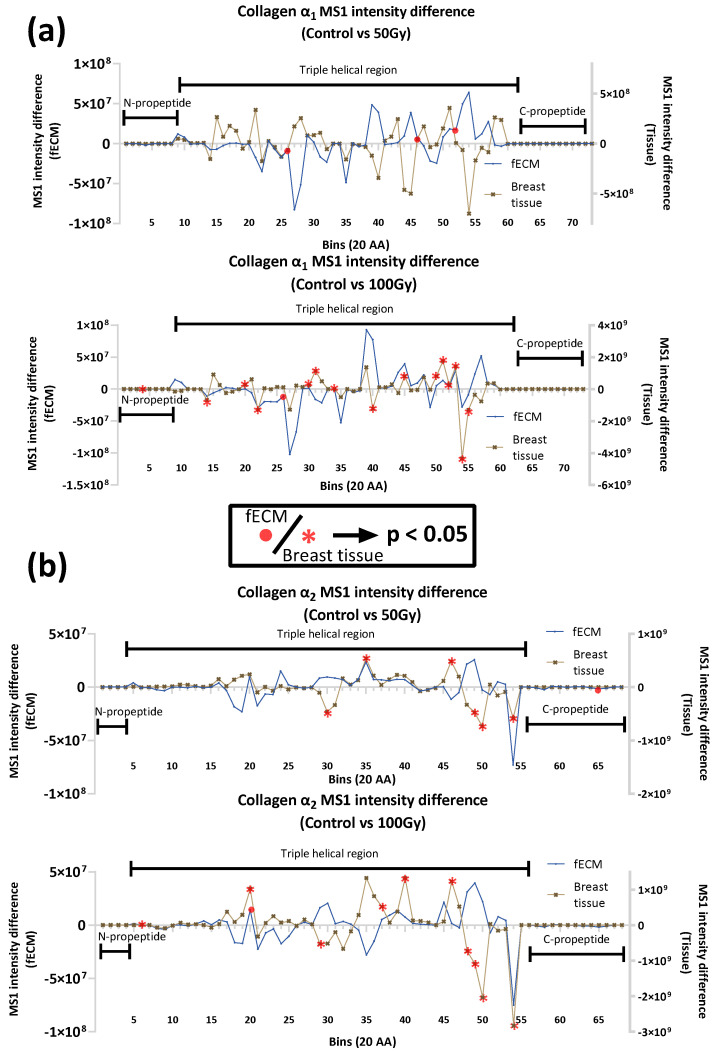

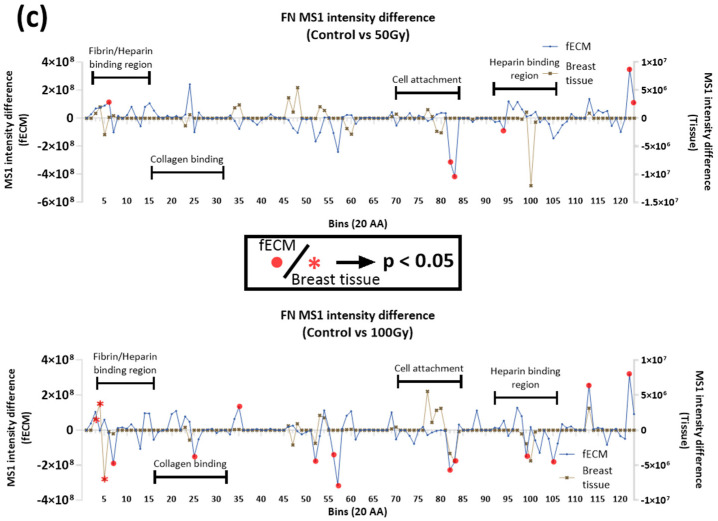
Peptide location fingerprinting analysis of collagen I and FN. Peptide location fingerprinting (PLF) analysis (see Figure 3 for detailed description of graph) was applied to LC-MS/MS data for collagen I α1 and α2 chains (**a**,**b**) and FN (**c**) in unexposed, 50 Gy- and 100 Gy-exposed fECM, and breast tissue samples. Statistically significant differences in peptide yield (unexposed control vs exposed) are highlighted in red.

Collagen I and FN were detected in both fECM and breast tissue samples and, as assessed by PLF, were susceptible to X-ray exposure. In both proteins, there were multiple regions with altered peptide yields, indicating changes in protein structure (Figure 5). The maturity and/or higher order assembly state of collagen I may be a key mediator of the extent and localisation of damage. With the exception of the 50 Gy X-ray-exposed α1 chain in fECM, statistically significant differences in peptide yield were predominantly detected in breast tissue collagen I (both α1 and α2 chains). The only region where X-ray exposure induced the same change in peptide fingerprint for both fECM and breast tissue collagen I was at bin 20 (aa. 380–400 of α2 chain exposed to 100 Gy).

Whilst FN X-ray susceptibility also differed between fECM and breast tissue, newly synthesised fECM FN accumulated more localised changes in peptide yield than breast tissue FN, and there was a clear dose dependence with an increase in the number of regions with altered peptide yield with the higher 100 Gy dose. In FN, affected regions detected at 50 Gy were close to fibrin/heparin-binding sites and to cell attachment regions. At 100 Gy exposure, peptide yield differences were also noted at the collagen-binding site.

In general, therefore, the effects of X-ray exposure on the peptide location fingerprints of collagen I and FN differ depending on the protein source—purified protein suspensions, fECM-derived, and breast tissue-derived. While collagen α chains in both fECM and breast tissue had PLF-flagged regions (indicating changes in proteolytic susceptibly), there was no clear consistency in the number of regions affected nor the positions of those regions. FN appeared to be more susceptible to X-rays in fECM samples, particularly at 100 Gy exposures, compared to breast tissue. We also observed a conserved change in peptide yield for the cell attachment region for both sources of FN exposed to 100 Gy (bins 82–83).

#### 2.2.4. Impact of X-Ray on Basement Membrane Proteins in Complex Matrices

**Figure 6:**
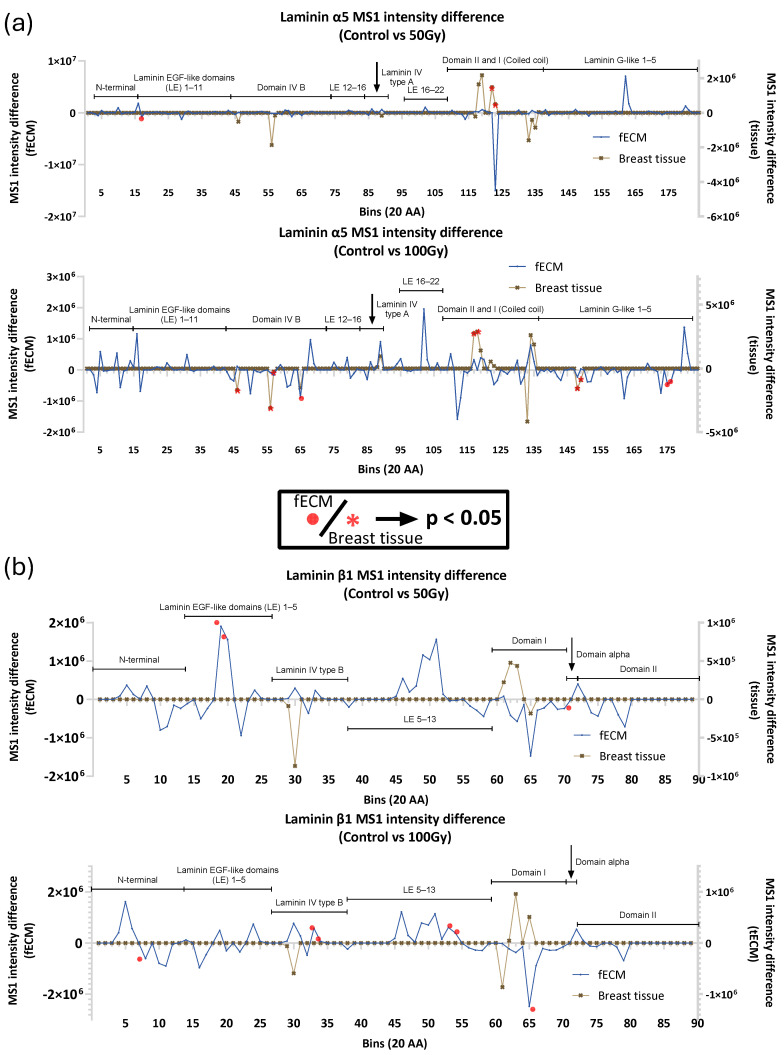
PLF analysis of laminin α5 and β1. (**a**) Changes in peptide yield in the laminin α5 chain in breast tissue were identified in domains I, II, IV. B and G-like domains were also affected. In fECM peptide yields were only affected at the higher 100 Gy dose. (**b**) The β_1_ chain from fECM exhibited changes in peptide yield in the EGF and alpha domains at 50 Gy and laminin IV type B domain at 100 Gy. No effects of X-ray exposure were observed in breast tissue laminin β_1_.

**Figure 7:**
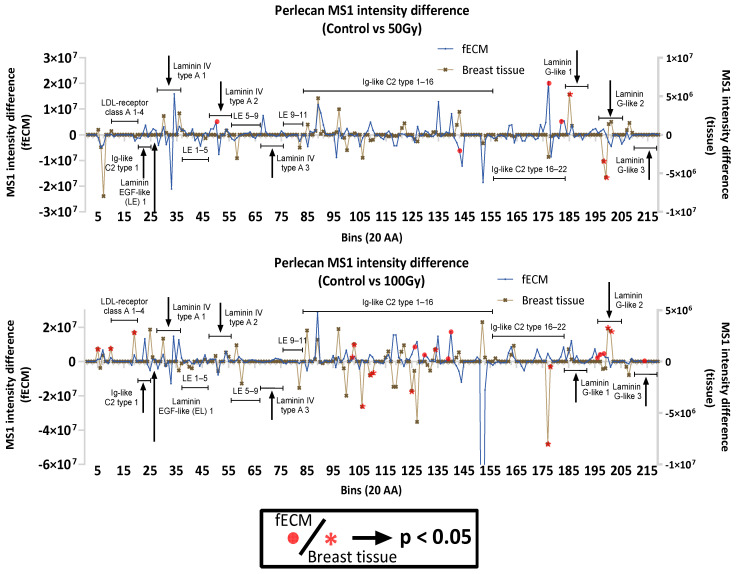
PLF analysis of perlecan. PLF analysis of perlecan identified multiple changes in peptide yield, which were dose-dependent.

In addition to collagen I and FN, other ECM proteins within fECM and breast tissue had significant changes in regional peptide yield as a consequence of X-ray exposure. In particular, the basement membrane proteins laminin (α5, β1) and perlecan were affected in both. Laminins α5 and β1 are constituents of laminin-511, a crucial basement membrane complex, which, in breast tissue, is a potent adhesive substrate for breast carcinoma cells [36,37]. Laminin α5 appears to be more X-ray-susceptible in breast tissue with significant changes in peptide yield in domain I and II at both 50 Gy and 100 Gy exposures and in domain IV and laminin G-like domains at 100 Gy (Figure 6). Conversely, laminin β1 seems to be more X-ray-labile in fECM with peptide yield changes in the laminin EGF-like domains. Multiple regions with significant differences in peptide yield were identified in both breast tissue (14) and fECM (8)-derived perlecan (Figure 7).

### 2.3. X-Ray Exposure Alters the Structure of Intracellular Proteins in Breast Tissue

**Figure 8:**
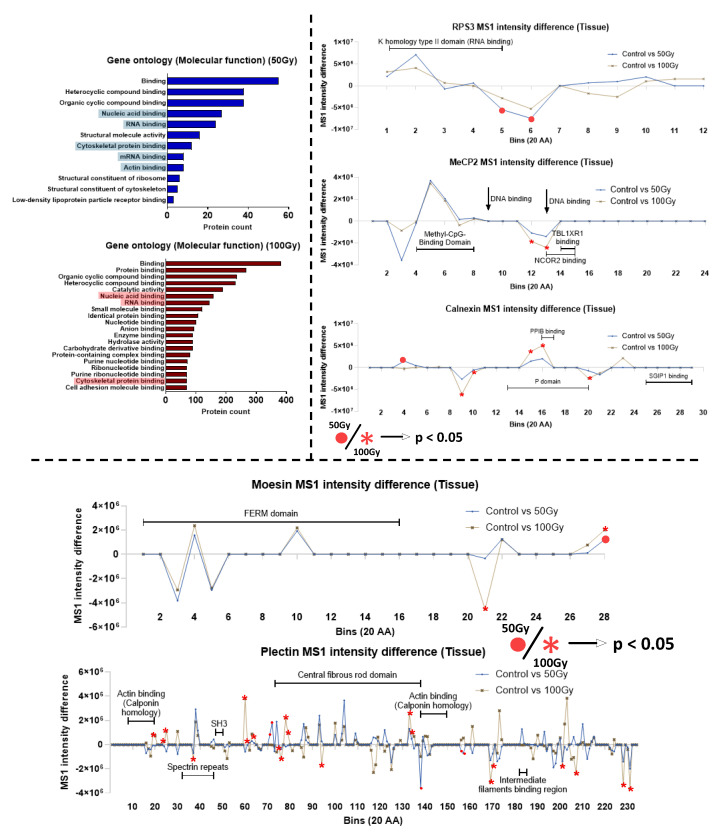
Impact of X-ray exposure on intracellular proteins in breast tissue. (**a**) Gene Ontology and functional analysis of intracellular proteins from breast tissue flagged by PLF consistently indicated the presence of cytoskeleton-binding and RNA-binding proteins. Exemplar proteins from the intracellular proteins flagged by PLF include 40S ribosomal protein S3 (RPS3), Methyl-CpG-binding protein 2 (MeCP2), Calnexin, Moesin, and Plectin, which were chosen based on their biological importance within the GO groups highlighted. (**b**) PLF analysis of RPS3 and MeCP2 indicated regions surrounding the RNA- and DNA-binding sites could be compromised. In Calnexin, PLF identified peptide yield differences in the P domain, which plays a role in regulating proper protein folding in the endoplasmic reticulum (ER). (**c**) PLF analysis of moesin identified non-significant but consistent changes (both 50/100 Gy) in the FERM domain. Significant peptide yield changes were found in the C-domain, which is an F-actin-binding site that also self-associates with the FERM domain. PLF analysis of plectin revealed multiple regional changes in peptide yields for regions near actin-binding regions after 100 Gy of X-ray exposure. Regions within the rod domain were also impacted.

Intracellular proteins were identified in breast tissue (which was not subjected to decellularisation and ECM enrichment procedures). Out of 671 intracellular proteins, 345 (51.4%) were highlighted by PLF with statistically significant regional changes to peptide yields. Gene ontology and functional analysis highlighted specific protein groups, including ribosomal and cytoskeleton proteins that appear to be X-ray-labile (Figure 8). Exemplar proteins were chosen from each protein group for PLF analysis, including 40S ribosomal protein S3 (RPS3), calnexin, and methyl-CpG-binding protein 2 (MeCP2) (Figure 8 and Supplementary Figures S2 and S3). RPS3 was chosen for its involvement in protein translation and DNA repair [38]. PLF analysis identified altered peptide yields for 100 Gy-exposed breast tissue within the K homology type II domain that is involved in RNA binding [39]. MeCP2 functions by binding to methylated DNA to promote or repress genes, affecting gene expression [40]. Peptide yield changes were identified in regions of the transcription repressor domain, where it recruits repressor proteins to repress the translation of genes. Calnexin functions as a protein folding checkpoint in the endoplasmic reticulum by binding to improperly folded N-linked glycoproteins [41] and potentially to misfolded procollagen I for ER-phagy [42]. Peptide yield changes were found in the p-domain, which is essential in recruiting other proteins (such as cyclophilin B) to assist in the folding of glycoproteins [43].

Cytoskeletal proteins affected by X-ray exposure include moesin and plectin. Moesin is part of the band 4.1 superfamily (protein 4.1, Ezrin, Radixin, and Moesin) that is involved in cellular cortex organisation and functions as a link between the cellular actin cytoskeleton to the membrane [44,45]. PLF analysis indicated changes to peptide yields in the FERM domain and in the C-terminal near to regions associated with F-actin binding. Finally, plectin stabilises the intermediate filament network by binding to different types of intermediate filament proteins [46]. This binding is crucial to stabilise the cytoskeleton of the cell and allow mechanosensing, such as the cross-talk between focal adhesions and intermediate filaments [47]. Numerous regions were highlighted by PLF to be impacted by X-rays, with much more in 100 Gy- than 50 Gy-exposed tissue. In 100 Gy-exposed samples, many impacted regions appear within the central rod domain. Regions surrounding the actin-binding sites also appear to be affected.

## 3. Discussion

### 3.1. Collagen I and Plasma Fibronectin Exhibit Differential Susceptibility to X-Rays

#### 3.1.1. Impact of X-Ray Exposure on Collagen I

Here, we confirm our previous observations [29] that X-ray doses of 50 Gy induce stochastic fragmentation, exhibited by the smearing pattern, which is similar to that produced chemically by oxidative mechanisms [48]. This suggests that radiation induced reactive oxygen species contribute to the cleavage of the peptide backbone. In the clinical context, these findings point to a potential mechanism by which radiation contributes to long-term tissue damage, such as fibrosis or ECM stiffening, by increasing collagen fragmentation and promoting subsequent degradation by MMPs [49,50,51]. The release of matrikines through such degradation, such as proline–glycine–proline (PGP) fragments [22,23], may have downstream effects on neutrophil recruitment and inflammation [52]. Additionally, the destabilisation of collagen I fibrils could also impact mechanosensitive pathways through changes in the stiffness of the cellular substrate [53,54]. This suggests a possible complex interplay between radiation-induced damage to the ECM and the immune response.

#### 3.1.2. Impact of X-Ray Exposure on Fibronectin

In contrast to collagen I, plasma FN exhibited greater resistance to fragmentation by X-rays. Radiation-exposed pFN had no observable changes in electrophoretic mobility from SDS-PAGE. This was further confirmed by differential scanning fluorimetry, revealing no significant changes in structure or thermal stability (Supplementary Figure S4), even at 100 Gy. The resilience of pFN to X-rays may be attributed to its globular conformation, which protects susceptible hydrophobic residues from oxidative damage [55,56]. Instead, ROS are directed to amino acid side chains on the surface of the protein. Given that ROS can alter protein structure, PLF analysis was employed to reveal potential changes to the structure (inferred from changes in peptide yield in LC-MS/MS data), which were localised to binding regions for key ECM components fibrin, heparin, fibulin, and collagen after 100 Gy of X-ray exposure. These protein-binding sites are often at the surface of the protein [57], suggesting that X-rays could be indirectly oxidising and damaging amino acids on the surface, which would be less prone to alter protein shape, compared with the oxidation of amino acids within hydrophobic regions [58,59].

These localised changes in peptide yield could play a role in the significant increase in irradiated FN’s binding affinity for collagen I, as confirmed by ELISA. We hypothesise that this increase in binding may be facilitated by the crosslinking of two synergistic collagen-binding regions (FNI_6_-FNII_1–2_FNI_7_, and FNI_8–9_) [60], which would reduce trypsin accessibility to the region (thus reducing peptide yields, as observed) but improve collagen binding, as they function in close proximity [61]. Such enhanced interactions of FN with collagen I may disrupt normal collagen fibrillogenesis [62]. FN plays a transient role in the initiation of collagen fibril formation [63]; enhanced binding could impede the proper nucleation and release of collagen I molecules, ultimately impairing ECM assembly. Although direct evidence for collagen I is lacking, previous studies on collagen III support this argument, showing that a high binding affinity of FN can negatively impact collagen III fibrillogenesis [64]. In turn, this could lead to abnormal tissue mechanics, translating to changes in cellular behaviour through mechanosensitive biochemical pathways, including Yes-associated protein-1 (YAP)/transcriptional coactivator with PDZ-binding motive (TAZ) [53], RhoA/rho-associated coiled-coil forming kinase (ROCK) [65], and the Integrin-mediated activation of focal adhesion kinase (FAK) to PI3K/AKT pathways [66].

These findings highlight a nuanced role for FN in the ECM’s response to radiation. While it is structurally resilient, its function as an ECM organiser may be compromised by radiation, leading to long-term effects on tissue mechanics.

#### 3.1.3. Radiation Impact on ECM Proteins in Complex Environments

Our analysis of more complex ECM environments, including both in vitro ECM-enriched fibroblast cell culture (fECM) and ex vivo breast tissue, revealed that X-ray exposures similar to that achieved in clinical settings can impact regional peptide yields of multiple ECM proteins in addition to collagen I and FN. These include key basement membrane proteins laminin and perlecan, which play critical roles in maintaining tissue integrity and mediating cell–ECM interactions.

The specific response of collagen I and FN in both fECM and breast tissue to X-rays was further compared to the changes found when exposed in purified solution. Whilst X-ray exposure affected collagen I and FN regardless of source (fECM or breast tissue), the localisation and extent of the induced damage differed. We have previously shown that collagen I is differentially susceptible to X-ray damage dependent on source (purified monomer, reconstituted fibrils, and tendon) [29]. Here, we show that newly synthesised collagen I differs in X-ray susceptibility to mature tissue collagen I, which may be attributable to age [67] and/or to the increased complexity of ECM interactions in the tissue environment where collagen I binds proteins, such as decorin, FN, and periostin.

Laminin and perlecan were identified by PLF as being affected by X-rays in both fECM and breast tissue. Laminin-511 is biologically relevant in basement membranes, and its expression has been shown to correspond with breast cancer and metastases [37]. Among the subunits of laminin, α_5_ exhibited the most alteration in regional peptide yields after 100 Gy of X-ray exposure. In particular, laminin IV type B (L4b) domain, domain I and II (laminin coiled coil domain, LCC), and laminin G-like (LG) domains were affected in the α_5_ chain. The impacted LG domains, especially the LG 4–5 domains in the α_5_ chain, are important for binding to heparin and α-dystroglycan [68,69]. Laminin binding to α-dystroglycan is crucial for normal muscle function and has been implicated in conditions like muscular dystrophy [70]. Radiation-induced structural changes in those regions could have biological implications on muscle health. The L4b domain in laminin β_1_ subunit also showed changes in peptide yields; although, the impact and the biological functions of this domain is still not well understood [71]. For perlecan, PLF revealed that 50 Gy of X-ray exposure alters the peptide yield around its C-terminus, including Ig-like C2 domains (in fECM) and the C-terminal domain V containing the laminin G-like domains (in breast tissue). These domains can be endogenously cleaved by matrix metalloproteinases (MMPs) to produce the matrikine endorepellin [72], which is able to bind to cell integrins (such as α_2_β_1_ integrin on endothelial cells) and interact with growth factors like VEGF to promote tissue repair [72,73]. Radiation-induced changes in the peptide yields of the matrikine-producing domains may indicate underlying structural changes that could alter the production of these matrikines, resulting in abnormal cell behaviours that contribute to the pathophysiology of radiation-induced side effects.

### 3.2. Radiation Impact to Intracellular Proteins in Complex Tissues

In addition to ECM proteins, PLF analysis flagged several intracellular proteins in breast tissue. Functional analysis highlighted that gene expression, cytoskeleton, and endoplasmic reticulum (ER)-associated protein groups were preferentially affected by radiation exposure based on PLF analysis. Exemplar proteins chosen for further analysis were RPS3, MeCP2, moesin, plectin, and calnexin. Ribosomal protein RPS3 and transcriptional regulator MeCP2 are both crucial mediators of gene expression and exhibited significant changes to peptide yield in regions responsible for RNA and DNA binding. Possible changes in the efficiency of RNA binding may impact the protein translation machinery [74]; although, the exact biological consequence/endpoint is still unclear. Given that RPS3 has also been implicated as a negative regulator of DSB repair [38,75], changes to its DNA-binding capabilities could impact DSB repair. Understanding the impact of radiation exposure on RPS3 and MeCP2 may also help us understand better, in radiation exposed cells, the mismatch between altered protein expression and mRNA changes [76,77]. Current literature has also shown that therapeutic levels of ionising radiation can impact the cell cytoskeleton and cellular adhesion molecules [78,79]. Here, we find the cytoskeletal proteins moesin and plectin had regions of altered peptide yields. In moesin, these regions were identified in the F-actin-binding site in the C-terminal region, which is known to self-associate with its FERM domain in its inactive form. Functional changes to the F-actin-binding site could alter its interaction with FERM, affecting the biological activation of moesin [80]. On the other hand, plectin exhibited numerous changes in peptide yield in its central rod domain, which is responsible for the formation of α-helices to facilitate the oligomerisation of plectin [46,81]. If impacted, the rod domain may compromise the stability of the α-helix and thus binding between plectin molecules. The oligomerisation of plectin has been implicated in facilitating proper mechanotransduction between focal adhesions and intermediate filaments, and the alteration of this link could impact the mechanosensing of cells to its environment [47]. Finally, in the endoplasmic reticulum component calnexin, PLF analysis identified peptide yield changes in the p-domain. This domain is essential to the function of calnexin in recruiting proteins (such as cyclophilin B) to assist in the folding of glycoproteins [43]. X-ray-induced changes to this domain could impair calnexin function, allowing misfolded proteins, which are detrimental to cellular homeostasis, to persist [82].

While this study has successfully employed a wide array of techniques to identify the potential impact of X-rays on breast ECM proteins, it is not without limitations. First, only one ex vivo breast sample was utilised in this study, which is insufficient to capture possible biological variations. However, the observation that many proteins are X-ray-labile in both tissue- and fibroblast-derived matrices suggests that individual–specific differences in the targets of X-ray-induced damage may be minimal. Additionally, we cannot be certain that a localised change in peptide yield equates to X-ray-induced damage at the same place. Changes in peptide yield could be caused by X-ray-induced damage to remote regions in the primary sequence. Furthermore, peptide yields in LC-MS/MS can be impacted by other chemical changes to the protein/peptides that are not directly attributed to X-ray damage [83,84]. Complementary techniques will be required to confirm localised changes in the three-dimensional configuration of proteins, such as by comparing with in silico datasets (i.e., AlphaFold [85]), to visually investigate regions flagged by PLF for biologically relevant functional changes. Finally, no fractionation of doses was conducted; therefore, this study does not replicate the dose regimens employed in vivo in a clinical setting. Further work is needed to unravel how X-ray dose fractionation and the subsequent cellular interactions with an altered ECM may affect tissue biology in radiotherapy patients.

**Figure 9:**
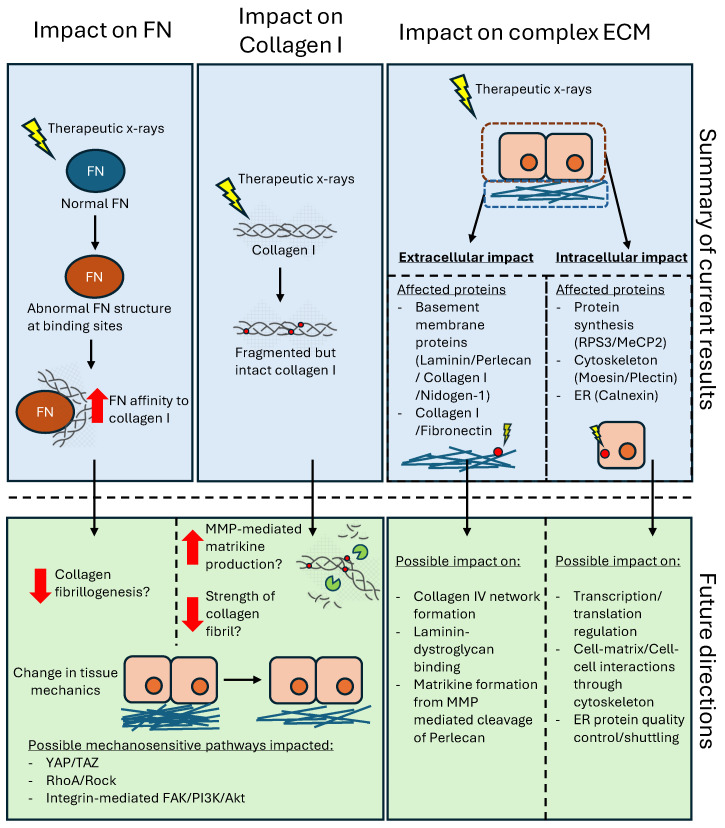
Potential consequences of breast tissue exposure to X-rays. This study highlights that the irradiation of FN increases its binding affinity to collagen I and also fragments collagen I. Further work will be needed to determine if this abnormal FN–collagen binding could decrease collagen I fibrillogenesis. Additionally, further investigation into how radiation-induced fragmentation of collagen I may increase its degradation by MMPs could elucidate the possible roles of matrikines in altering tissue mechanics. Finally, the characterisation of X-ray-induced changes to mechanosensitive pathways, such as YAP/TAZ, RhoA/ROCK, and integrin-mediated FAK/PI3K/Akt, could be beneficial in understanding the downstream pathways impacted by radiation damaged collagen and FN.

In conclusion, the results presented in this study demonstrate that total X-ray doses similar to those employed clinically, whilst effective in targeting cancer cells, can also induce significant structural changes in ECM proteins and intracellular components, with potential long-term consequences for tissue integrity and function. The observed alterations in collagen I and FN may contribute to altered tissue mechanics, while changes in basement membrane and intracellular proteins could exacerbate abnormal cellular dysfunction through biochemical cues or altered cell–ECM interactions (Figure 9). Future work could focus on exploring the mechanistic basis of these structural changes, and the subsequent biological consequences on cell–matrix interactions. Other higher resolution structural techniques, such as TEM or cryo-EM, may further provide insights on how radiation modifies specific protein conformation, while SPR would help decipher changes to protein–protein binding affinities.

## 4. Materials and Methods

### 4.1. Biological Materials

Human collagen I and purified plasma fibronectin (pFN) were both purchased from Abcam (catalogue numbers ab7533 and ab80021, respectively: Abcam, Cambridge, UK). Immortalised human mammary fibroblasts (HMFU-19) for in vitro ECM production were kindly supplied by Dr Andrew Gilmore, University of Manchester (at passage 18–20). HMFU-19 are normal primary human mammary fibroblasts immortalised by human telomerase reverse transcriptase (hTERT) and a temperature-sensitive mutant of the simian virus 40 large-tumour antigen (U19tsA58) [86]. Ex vivo frozen normal breast tissue samples from a 64-year-old female were obtained from the Manchester Cancer Research Centre Biobank under the Manchester Cancer Research Centre (MCRC) general ethics approval. The MCRC Biobank is licensed by the Human Tissue Authority (licence number: 30004) and has been ethically approved as a research tissue bank by the South Manchester Research Ethics Committee (Ref: 22/NW/0237: approved 30 August 2022: commenced 13/10/22). Human samples are obtained with informed consent.

### 4.2. X-Ray Irradiation

X-ray exposure for all sample types was conducted at room temperature in an Xstrahl C1X3 320kV cabinet irradiator (Xstrahl, Walsall, UK). The instrument yielded a dose of 2.2 Gy/min with 300 kVp tube voltage, 10 mA electron current, and a 0.7 mm Cu filter. Samples were irradiated to a total dose of 50/100 Gy X-rays, delivered in 22 min 16 s and 45 min 27 s, respectively. Purified protein suspensions (solubilised collagen I and pFN) were irradiated in Eppendorf tubes (with an estimated attenuation due to tube wall of 5%, as described previously [39]). Fibroblast-derived fECM samples were irradiated in 6-well plates under approximately 0.5 mL of PBS+ to prevent dehydration. Breast tissue samples were irradiated as cryosections on their glass slides. Five technical replicates (*n* = 5) were used for all conditions (irradiated samples and non-irradiated controls).

### 4.3. Protein Gel Electrophoresis of Purified Proteins

SDS-PAGE was conducted on gradient (4–12% Bis-tris NuPAGE) gels in 3-morpholinopropane-1-sulfonic acid (MOPS) SDS running buffer at 200 V for 60 min in an XCell surelock mini-cell electrophoresis system (ThermoFisher, Cambridge, UK). Subsequently, gels were stained with a Pierce silver staining for mass spectrometry kit (ThermoFisher, Cambridge, UK).

### 4.4. Fibroblast Extracellular Matrix Enrichment and Processing

HMFU-19 fibroblasts were cultured at passage 20–22 and grown in RPMI-1460 media, with 1% Glutamax and 10% FBS. Cells were seeded in 6-well plates at a density of 10^5^ per dish/well and grown until confluent (2–3 days). Media was changed every 2 days, and cells were allowed to deposit matrix until 9 days post-confluence. The matrix was decellularised following previously established protocol [87,88] by incubating with PBS− (137 mM NaCl, 2.7 mM KCl, 10 mM Na_2_HPO_4_, 1.8 mM KH_2_PO_4_, pH 7.4) (Sigma-Aldrich, Dorset, UK) containing 0.5 mL of 20 mM NH_4_OH, 0.005% Triton-X 100 until no cells were visible. An additional 1 mL of PBS− was added to each well incubates overnight at 4 °C to allow cells to detach fully. Plates/wells were carefully washed twice with PBS− and twice with PBS+ (PBS with 0.9 mM CaCl_2_ and 0.5 mM MgCl_2_) to remove detergent and remaining cells. The resultant decellularised, ECM-enriched sample (fECM) was then stored in PBS+ supplemented with 1% penicillin/streptomycin at 4 °C (up to 5 days) until use.

**Figure 10:**
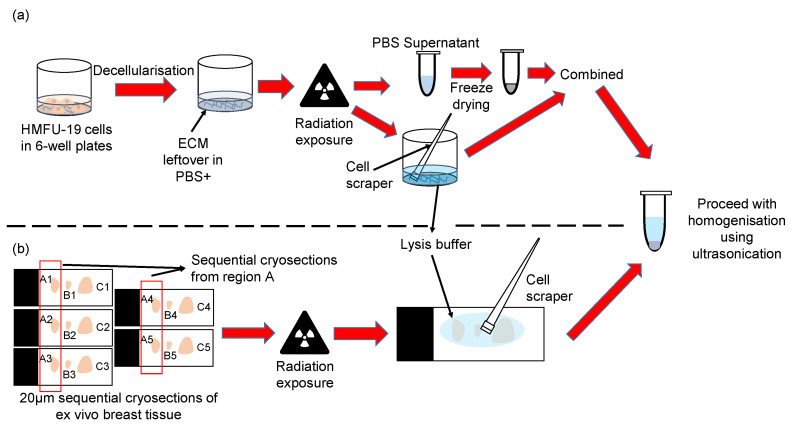
Protein extraction and homogenisation steps for in vitro HMFU-19 cell culture and ex vivo breast tissue samples. (**a**) Following decellularisation, the attached material in PBS was exposed to radiation (*n* = 5). The supernatant was then collected and freeze-dried, whilst the ECM was scraped from the wells into SDS-containing lysis buffer. The lyophilised supernatant was resuspended in the same lysis buffer and combined with the ECM fraction before being homogenised with ultrasonication. (**b**) An ex vivo normal breast tissue sample from a 64-year-old was cryosectioned into sequential sections of 20 μm thickness (e.g., A1 to A5 are sequential sections from a region of tissue; B1 to B5 are sequential sections from a different region of the same tissue). Five sequential sections were obtained for each region of the tissue, to a total of 9 regions, with 3 regions combined into a slide. This gave a total of 3 sets of 5 = 15 slides. Within each set of slides, the first and last slides were kept for H&E staining, while the 3 remaining slides were randomly chosen to be exposed to 0, 50, or 100 Gy of radiation. After radiation exposure, OCT was removed by a quick ethanol wash, and subsequently, the sections were scrapped off the slides with a cell scrapper and addition of lysis buffer. The detached sections were then ultrasonicated.

Fibroblast derived samples (fECM) were processed for LC-MS/MS analysis, as follows (Figure 10). The supernatant (PBS+ containing penicillin/streptomycin) was collected to prevent loss of protein fragments. A total of 400 μL of lysis buffer was added to the decellularised ECM in 6-well plates. Following this, the ECM was removed with the buffer and collected into an Eppendorf tube using a mini cell scraper (VWR, Radnor, PA, USA). The supernatant was frozen down in −20 °C freezer and lyophilised in a freeze dryer overnight. Subsequently, 100 μL of lysis buffer was added to the lyophilised supernatant and combined with the ECM samples to a total volume of 500 μL. The samples were then homogenised with ultrasonication using Covaris LE220+ (Covaris LLC, Woburn, MA, USA) at 500 W peak power, 20% duty factor (100 W average) for 5 min. Protein concentration was subsequently determined by Direct Detect™ (Merck Millipore, Darmstadt, Germany) assay before storage at −20 °C.

### 4.5. Breast Tissue Preparation and Processing

An ex vivo normal breast tissue sample from a 64-year-old patient was cryosectioned prior to X-ray irradiation. Frozen tissue was embedded in Tissue-tek optimal cutting temperature (OCT) compound (Sakura Finetek, Torrance, CA, USA) and sectioned to a nominal thickness of 20 µm. (Each section contained sufficient tissue for proteomic analysis whilst retaining similar tissue composition (by H&E staining) across five sequential sections (Supplementary Methods).) These five sequential sections were collected on individual Epredia Superfrost™ microscope slide (ThermoFisher, UK) to form a group. Each slide contained three cryosections from different groups but of the same sequential position in their respective group (Figure 10). Slides were kept at −20 °C and transported on dry ice until X-ray exposure.

Breast tissue cryosections (both irradiated and non-irradiated) were processed to remove the OCT compound. Slides were washed in 70% EtOH then 100% EtOH for 30 s each. EtOH was then removed by ten washes (five in each of two containers) of deionised H_2_O. The slides were then given the same EtOH treatment (70% EtOH 30 s, 100% EtOH 30 s) to remove any residual water and OCT and left to dry for 30 min at room temperature. Adhered cryosections were then rehydrated with approximately 200 μL of lysis buffer (5% SDS, 50 mM triethylammonium bicarbonate (TEAB), pH 7.5) and carefully loosened from the slides using a mini cell scraper (Figure 10). The material was then transferred over to a microTUBE-500 (Covaris LLC, Woburn, MA, USA). Additional drops of lysis buffer were added to the slides to collect as remaining material and subsequently combined in the microTUBE-500. The total volume made up to 500 μL with lysis buffer. Samples were ultrasonicated in a Covaris LE220+ (Covaris LLC, Woburn, MA, USA) at 500 W peak power, 20% duty factor (100 W average) for 5 min. Protein concentration was subsequently determined by Direct Detect™ (Merck Millipore, Darmstadt, Germany) assay, before storage at −20 °C.

### 4.6. Mass Spectrometry Sample Preparation

LC-MS/MS was employed to compare protein identity, protein abundance, and potential structural differences (by PLF) in non-irradiated (control) and irradiated samples. Sample preparation for LC-MS/MS was via established protocols [29,30,31]. Briefly, for each sample, 10 μg of purified commercial protein or 100 μg of homogenised fECM or breast tissue extract (protein amount determined by Direct Detect method) was diluted to either 130 μL (purified proteins) or 500 μL (fECM and breast tissue in 5% SDS, 50 mM triethylammonium bicarbonate (TEAB), pH 7.5, prior to reduction with 5 mM Dithiothreitol (DTT) and alkylation with 15 mM Iodoacetamide (IAM)). Phosphoric acid was added to a final concentration of 1.2% (*v*/*v*), followed by protein precipitation with binding buffer (100 mM TEAB, 90% Methanol, pH 7.1) at a ratio of 6:1 (buffer: sample) (*v*/*v*). Proteins were then concentrated by an S-trap column (ProtiFi LLC, Fairport, NY, USA), which was washed with binding buffer before the addition of 20 μL trypsin at a ratio of 1:10 trypsin: sample in 50 mM TEAB. Trypsin digestion was at 37 °C for 16–24 h.

Digested peptide suspensions were sequentially eluted with 50 mM TEAB, 0.1% (*v*/*v*) aqueous formic acid (FA) and finally 0.1% (*v*/*v*) FA in 30% (*v*/*v*) acetonitrile to a final 5% (*v*/*v*) acetonitrile concentration. Samples were desalted and washed with 0.1% formic acid using Oligo R3 beads added to 96-well Corning FiltrEX desalt filter plates. Next, peptides were eluted with 0.1% formic acid in 30% (*v*/*v*) acetonitrile and dried with Heto speed vacuum concentrator centrifuge (ThermoFisher, UK) without heating for 90 min. Vacuum-dried peptide samples were then stored at 4 °C and resuspended in 5% (*v*/*v*) acetonitrile in 0.1% (*v*/*v*) FA prior to LC-MS/MS.

### 4.7. Mass Spectrometry Sample Runs

LC-MS/MS was performed following the protocol outlined by Eckersley (2018) [89]. Briefly, the experiment utilised an UltiMate^®^ 3000 Rapid Separation LC system (RSLC, Dionex Corporation, Sunnyvale, CA, USA) coupled to an Orbitrap Exploris 480 (ThermoFisher, UK) (for pFN and fECM) or Orbitrap Fusion Lumos Tribrid mass spectrometry system (for breast tissue samples). The mobile phases consisted of buffer A (0.1% formic acid in water) and buffer B (0.1% formic acid in acetonitrile). A 2 μL aliquot of resuspended peptides (100 ng/μL) was injected into a nanoEase M/Z Peptide CSH C18 analytical column (130 Å, 1.7 µm, 75 µm × 250 mm, Waters Corp, Milford, MA, USA) at a flow rate of 300 nL/min. Peptides were separated with a multistage gradient, as follows: 1% to 6% buffer B over 2 min, 6% to 18% buffer B over 44 min, 18% to 29% buffer B over 7 min, and 29% to 65% buffer B over 1 min. MS2 data acquisition was performed in a data-dependent mode, covering a scan range of 300–1750Th.

### 4.8. Mass Spectrometry Protein Identification and Quantification

Proteome Discoverer 2.5 (ThermoFisher, UK) was used for protein identification with a pre-optimised workflow, as previously described [29]. Spectrum searches were conducted using SEQUEST within Proteome Discoverer. The SwissProt database (retrieved from UniProtKB, release 2022_01) was used to search for fully tryptic peptides, with *Homo sapiens* specified as the taxonomy. Search parameters include a pre-cursor ion tolerance of 10 ppm and an MS/MS fragment ion tolerance of 0.2Da. Fixed modifications considered included the carbamidomethylation of cysteine residues, while variable modifications considered were the oxidation of lysine, methionine, proline, and arginine. A maximum of two missed cleavages and peptide charge states of +1, +2, and +3 were considered. The peptide false discovery rate was controlled at 1%. Identified peptides lacking pre-cursor ion (MS1) intensity quantification were excluded. Only peptides uniquely mapped to a single protein were included in the analysis. The analysed peptide data were normalised abundances, which were used for MS1 intensity quantification mapping in PLF analysis. Protein abundances were compared using total peptide spectrum matches (PSMs) as a proxy. All data were exported to an excel file for post-processing using Python 3.10.9.

### 4.9. Peptide Location Fingerprinting Analysis

The peptide location fingerprinting (PLF) analysis of LC-MS/MS data was used to identify regions in proteins with significant differences in localised trypsin-mediated cleavage and hence peptide yields [30,31]. Such differences may be due to radiation-induced structural changes, which in turn, may affect function. A custom python program (https://github.com/RJTuieng/MS1_PLF.git (accessed on 9 June 2025) was developed to enhance PLF analysis, as previously described [29,31]. In brief, peptide sequences, their respective MS1 intensities, and master protein accession were obtained from the Proteome Discoverer exported data. Peptides were grouped by their master protein, and the peptide’s MS1 intensity is mapped onto the amino acids in their respective protein’s sequence. After mapping, each protein sequence was divided into 20 amino acid bins, with any residual amino acid combined into the last bin. The MS1 intensities of the amino acids in each bin were averaged to determine the bin’s MS1 intensity. Subsequently, the MS1 intensities of all bins across the entire protein sequence were then normalised by multiplying with a normalisation factor. This factor is derived by taking the sum of all peptide MS1 intensities of the protein in that sample group and dividing it from the sum of all peptide MS1 intensity of the sample with the highest total MS1 intensity of that protein. This forms the peptide location fingerprint of the protein. For each protein, PLF data were exported from python to GraphPad Prism 9.0 (GraphPad Software, Boston, MA, USA). Two-way repeated-measures ANOVA with Geisser–Greenhouse correction and Bonferroni-corrected multiple comparisons was used for testing statistical significance. All sample groups were analysed in replicates of five.

### 4.10. Solid Phase Enzyme-Linked Immunosorbent Assay for Collagen–pFN Binding

This assay was conducted to test for protein–protein binding between purified human collagen I and human pFN and is adapted from a previously published protocol [90]. In brief, human collagen I (ab7533, Abcam, Cambridge, UK) was diluted to 2.5 μg/mL using PBS− (Sigma-Aldrich, UK) and added to 96-well plates at 50 μL/well for overnight coating at 4 °C. Plates were washed three times (with 0 min, 2 min, 3 min incubations) with 25 mM Tris, 2.7 mM KCl, 137 mM NaCl, 0.05% Tween-20, pH 7.4 (prepared from 20X TBS (ThermoFisher, UK) and Tween-20 (Sigma-Aldrich, UK)) (TBST), then blocked with 200 μL/well of 5% skim milk (SERVA Electrophoresis GmbH, Germany) in TBST at room temperature (RT) for 1.5 h. Following the same wash procedure, plasma FN (ab80021, Abcam), which was diluted to a range of 20–0.05 μg/mL in 20 mM HEPES, 150 mM NaCl, was added at 50 μL/well and incubated overnight at 4 °C. Subsequently, the plasma FN solution was removed from the plates by washing with TBST, before the addition of FN mouse monoclonal 1° antibody (66042-1-Ig, Proteintech Europe, UK) at 1:2000 with 100 μL/well for overnight incubation at 4 °C. The primary antibody was washed out with a stricter wash procedure (5× wash: 0 min, 2 min, 2 min, 3 min, 3 min incubations), before the addition of the 2° antibody (HRP-conjugated Affinipure Goat anti-rabbit IgG(H + L), SA00001-2, Proteintech Europe, UK) at 1:2000, 100 μL/well, for 1.5 h incubation at RT. Finally, following a strict 5× wash with TBST, 100 μL of 3,3′,5,5′-tetramethylbenzidine (TMB) ELISA substrate (Highest sensitivity) (ab171522, Abcam, Cambridge, UK) was added per well and incubated for 40 min. The reaction was stopped by adding equal volume (100 μL) of 1 M HCl to the wells. The absorbance was recorded at 450 nm with Multiskan FC (ThermoFisher, UK).

### 4.11. Statistical Analyses

A Student’s *t*-test was applied for statistical testing for band/regional intensities in SDS-PAGE. For PLF analysis, two-way repeated measures ANOVA with Geisser–Greenhouse correction and Bonferroni-corrected multiple comparisons test was used. *p* value for significance was set at 0.05 for all statistical tests.

## Data Availability

The mass spectrometry proteomics data have been deposited to the ProteomeXchange Consortium via the PRIDE partner repository. Data can be accessed via PRIDE (https://www.ebi.ac.uk/pride (accessed on 9 June 2025)) with the following DOI: human fibroblast-derived ECM (DOI: 10.6019/PXD060843), breast tissue (DOI: 10.6019/PXD060871), solubilised plasma fibronectin (DOI: 10.6019/PXD060917).

## Supplementary Materials

The following supporting information can be downloaded at https://www.mdpi.com/article/10.3390/ijms26125696/s1.

## Abbreviations

The following abbreviations are used in this manuscript:

DSB	DNA double-strand break
DTT	Dithiothreitol
ECM	Extracellular matrix
ELISA	Enzyme-linked immunosorbent assay
ER	Endoplasmic reticulum
FAK	Focal adhesion kinase
fECM	ECM-enriched sample from cultured fibroblasts
FN	Fibronectin
H&E	Hematoxylin and eosin
HMFU-19	Normal primary human mammary fibroblasts
hTERT	Human telomerase reverse transcriptase
IAM	Iodoacetamide
LCC	Laminin coiled coil domain
LC-MS/MS	Liquid chromatography with tandem mass spectrometry
LG	Laminin G-like (LG) domains
MOPS	3-morpholinopropane-1-sulfonic acid
OCT	Optimal cutting temperature (OCT) compound
PGP	Proline–glycine–proline
PLF	Peptide location fingerprinting
pFN	Plasma fibronectin
ROCK	RhoA/rho-associated coiled-coil forming kinase
ROS	Reactive oxygen species
RT	Room temperature
SDS-PAGE	Sodium dodecyl sulphate–polyacrylamide gel electrophoresis
TAZ	Yes-associated protein-1 (YAP)/transcriptional coactivator with PDZ-binding motive
TEAB	Triethylammonium bicarbonate
TBST	Tris-buffered saline with Tween 20,
TMB	3,3′,5,5′-tetramethylbenzidine
