# Supplementary material for: X-Ray Exposure Induces Structural Changes in Human Breast Proteins
Source: Int J Mol Sci. 2025 Jun 13;26(12):5696. doi: 10.3390/ijms26125696 (PMC12193695; doi:10.3390/ijms26125696)
Supplement: Supplementary file 1 [file ijms-26-05696-s001.zip › ijms-3647081-supplementary.pdf]

Supplementary material:

X-Ray Exposure Induces Structural Changes in Human Breast Proteins

Ren Jie Tuieng [1,2], Sarah H. Cartmell [3], Cliona C. Kirwan [4,5], Alexander Eckersley [5,6] and Michael J. Sherratt [1,6*]

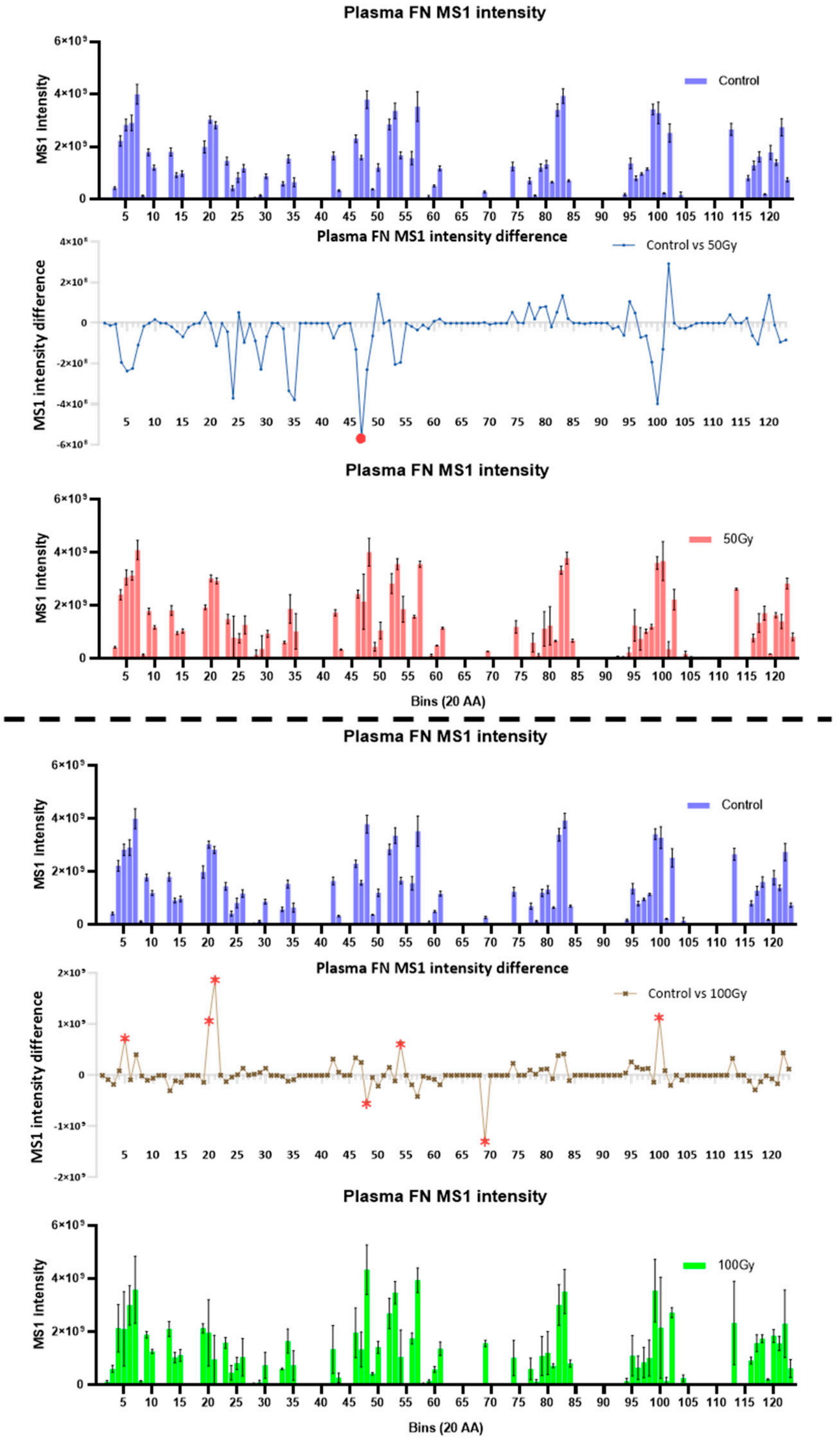

Figure S1. *Peptide location fingerprinting analysis for solubilised human plasma fibronectin. MS1 intensity difference graphs are sandwiched between the MS1 intensity peptide fingerprint graph of control (top) and x-ray exposed (bottom) samples respectively. A red icon is depicted where the region of the protein exhibit statistically significant differences in MS1 intensity between control and irradiated samples.*

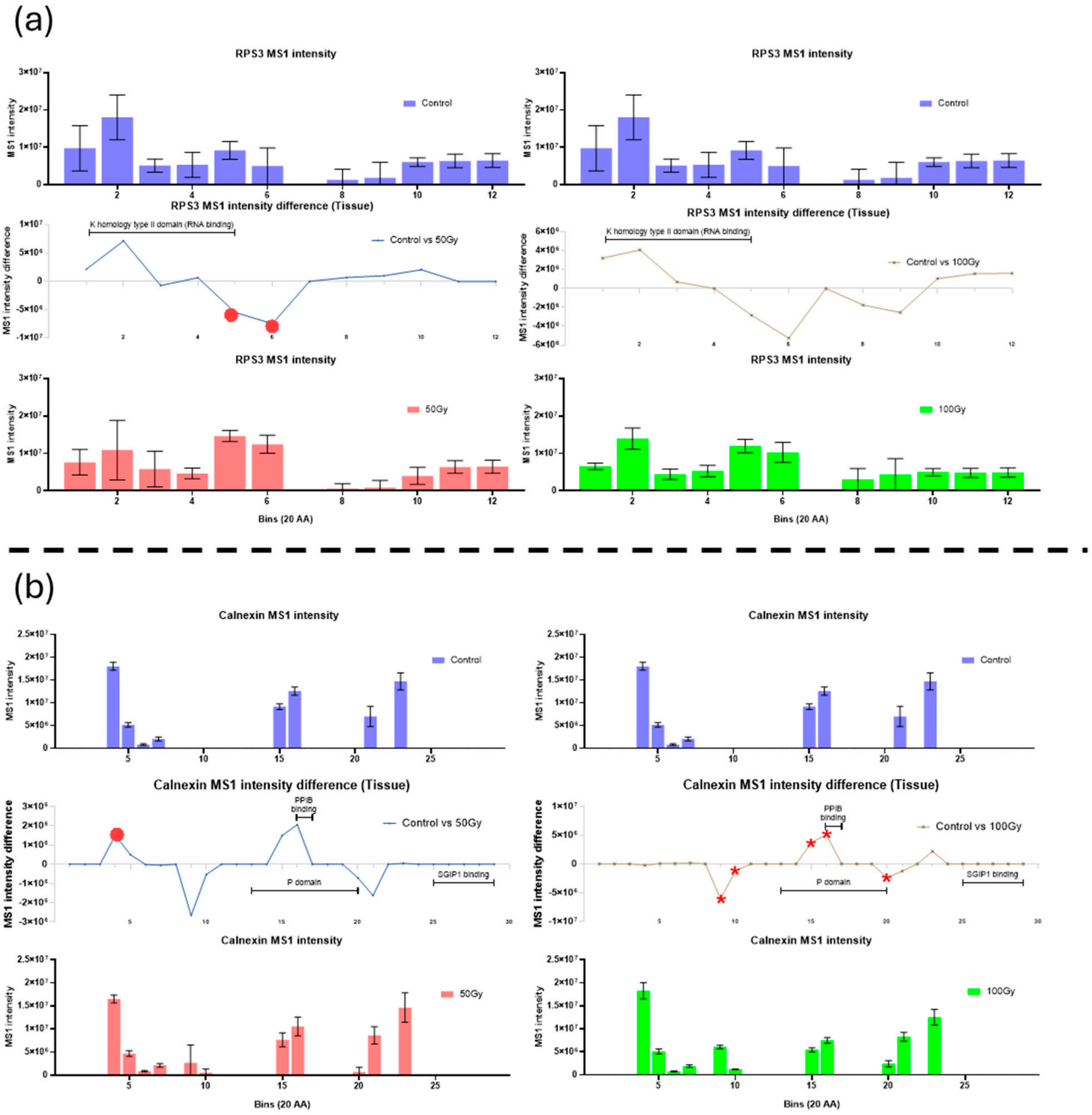

Figure S2. *Peptide location fingerprinting analysis for intracellular proteins (a) RPS3 and (b) Calnexin found in breast tissue. For each protein, MS1 intensity difference graphs are sandwiched between the MS1 intensity peptide fingerprint graph of control (top) and x-ray exposed (bottom) samples respectively. A red icon is depicted where the region of the protein exhibit statistically significant differences in MS1 intensity between control and irradiated samples.*

(a)

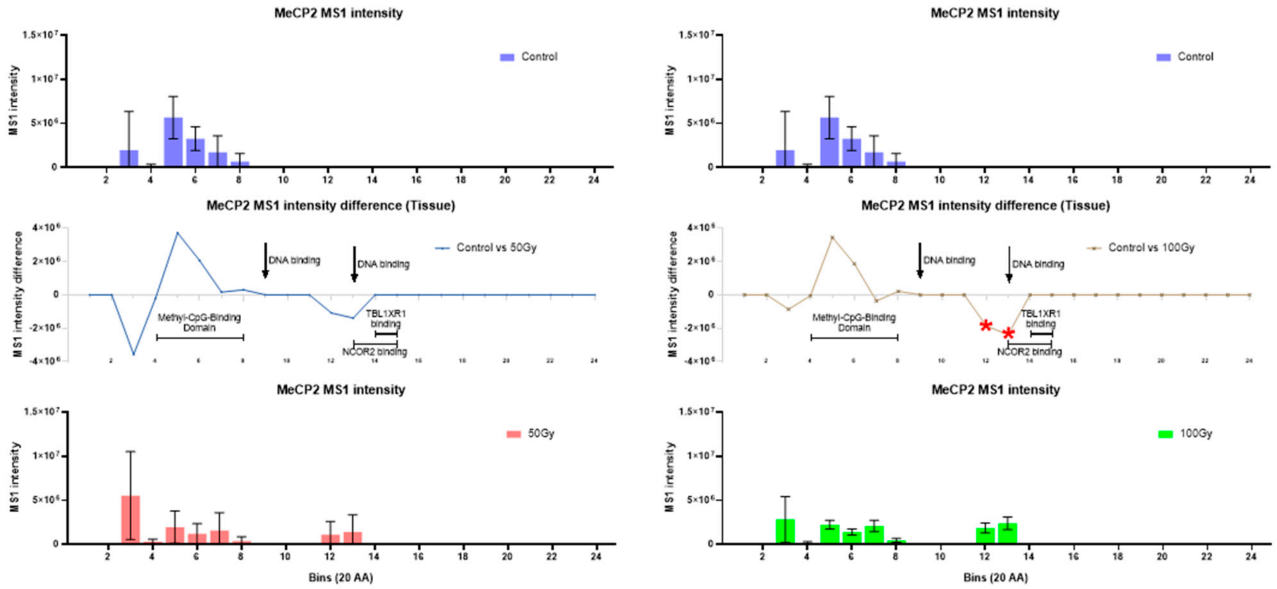

Figure S3. *Peptide location fingerprinting analysis for intracellular MeCP2 in breast tissue. MS1 intensity difference graphs are sandwiched between the MS1 intensity peptide fingerprint graph of control (top) and x-ray exposed (bottom) samples respectively. A red icon is depicted where the region of the protein exhibit statistically significant differences in MS1 intensity between control and irradiated samples.*

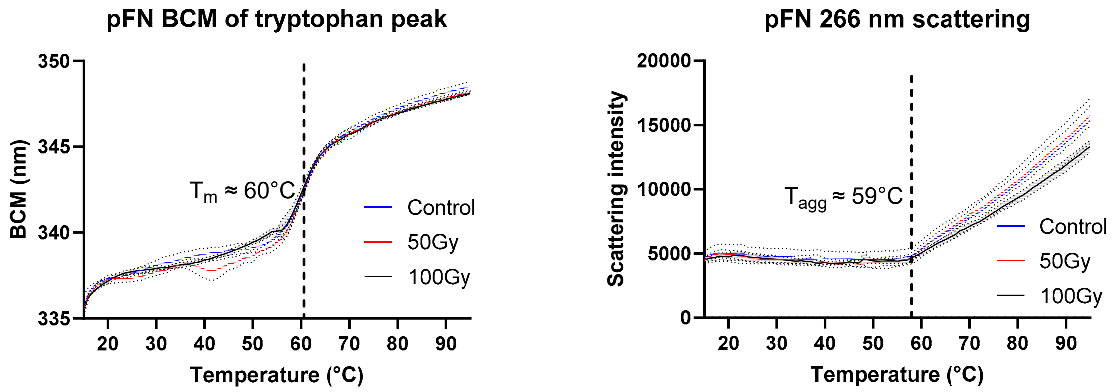

Figure S4. *Differential scanning fluorimetry of pFN found no change in the barycentric mean (BCM) of the tryptophan peak and 266nm scattering, indicating no change in melting and aggregating temperatures. This implies no significant changes in protein structure of pFN.*
